# Draft Genome Sequence of Bifidobacterium bifidum Strain ICIS-310, Isolated from the Feces of a Healthy 5-Year-Old Child from Orenburg, Russia

**DOI:** 10.1128/MRA.01271-18

**Published:** 2018-11-08

**Authors:** Sergey V. Andryuschenko, Elena V. Ivanova, Natalia B. Perunova, Irina A. Zdvizhkova, Anastasiya V. Bekpergenova, Oleg V. Bukharin

**Affiliations:** a Institute for Cellular and Intracellular Symbiosis of Ural Branch of Russian Academy of Sciences, Orenburg, Russia; University of Rochester School of Medicine and Dentistry

## Abstract

This report describes the draft genome sequence of Bifidobacterium bifidum strain ICIS-310, isolated from the feces of a healthy 5-year-old child from Orenburg, Russia. The size of the genome was 2,219,632 bp (62.4% G+C content). Annotation revealed 1,886 coding sequences, including 1,718 proteins, 6 rRNA genes, and 52 tRNA genes.

## Abstract (precis)

This report describes the draft genome sequence of Bifidobacterium bifidum strain ICIS-310, isolated from the feces of a healthy 5-year-old child from Orenburg, Russia. The size of the genome was 2,219,632 bp (62.4% G+C content).

## ANNOUNCEMENT

Bifidobacteria are important representatives of the obligate microflora of the human intestine and occupy the leading position among representatives of the indigenous microflora, forming the basis of colonization resistance of the host due to the production of antimicrobial substances and stimulation of the factors of innate and adaptive host immunity against pathogens (1, 2). The genus Bifidobacterium is a member of the Actinobacteria phylum and Bifidobacteriaceae family (3). B. bifidum can release biologically active substances in the intestine (4, 5) and interact with other intestinal microorganisms, resulting in colonization resistance (6); they produce vitamins and participate in the biosynthesis of amino acids, as well as in the exchange of minerals and microelements.

Here, we present a draft genome sequence of B. bifidum strain ICIS-310, isolated from the feces of a healthy 5-year-old child from Orenburg, Russia.

Preparation of DNA libraries and sequencing were conducted in the Center of Shared Equipment “Persistence of microorganisms” of the Institute for Cellular and Intracellular Symbiosis of the Ural Branch of the Russian Academy of Sciences (RAS; Orenburg, Russia).

Strain B. bifidum ICIS-310 was cultivated in 4 ml of Shaedler medium (HiMedia Laboratories Pvt. Ltd.) during 48 h in a 0.6% oxygen and 5% carbon dioxide atmosphere and at a temperature of 37°C in a CO_2_ incubator (Binder, Tuttlingen, Germany). After incubation, the culture was centrifuged at 4,000 × *g* for 6 min. The sediment was resuspended in 50 μl of Tris-buffered saline with 2 μg of lysozyme from chicken egg white and incubated at 37°C for 60 min. The suspension was mechanically homogenized by 1.4-mm silica beads at a speed of 6.5 m/s for 1 min. DNAses were inactivated by heating of suspension to 95°C for 10 min, and then 50 μl of 10% SDS solution and 2 μl of 100 mg/ml proteinase K solution were added to the suspension, with subsequent incubation at 60°C for 60 min. The extracted DNA solution was purified by a standard phenol-chloroform extraction method (7) and precipitated by ethanol (8). The DNA sediment was dissolved in 30 μl of Milli-Q deionized water.

The genomic DNA of B. bifidum ICIS-310 was used to prepare a DNA library with the Nextera XT DNA sample preparation kit (Illumina, San Diego, CA). The library was sequenced in a 2 × 300-nucleotide run using the MiSeq reagent kit version 3 and a MiSeq desktop sequencer (Illumina). The reads were quality trimmed using the sliding window mode of the Trimmomatic program (9). *De novo* genome assembly was performed using the SPAdes genome assembler (version 3.9.0) (10). The assembly yielded 32 contigs covering a total of 2,219,632 bp, with an *N*_50_ of 254,356, a G+C content of 62.4%, and an average coverage of 44.35×. Four contigs were less than 200 bp and were removed from the analysis. The genome sequence was annotated using the National Center for Biotechnology Information (NCBI) Prokaryotic Genome Annotation Pipeline (PGAP) (https://www.ncbi.nlm.nih.gov/genome/annotation_prok), which revealed 2,173 coding sequences, including 1,718 proteins, 107 pseudogenes, 6 rRNA genes (5S, 16S, and 23S), 52 tRNA genes, and 3 noncoding RNA (ncRNA) genes.

Analysis of the strain B. bifidum strain ICIS-310 genome revealed genes involved in resistance to antibiotics, such as ceftazidime, ciprofloxacin, lomefloxacin, azithromycin, and ampicillin. The ANAEROtest 24 biochemical method (Lachema, Czech Republic) revealed that the B. bifidum strain ferments glucose, fructose, galactose, melezitose, esculin, mannose, cellobiose, lactose, sucrose, salicin, trehalose, mannitol, rhamnose, arabinose, and sorbitol.

B. bifidum ICIS-310 can serve as a model strain for studies investigating symbiotic relations of bacteria and humans. In addition, the revealed properties of B. bifidum ICIS-310 may be crucial for further development of probiotics.

### Data availability.

This whole-genome shotgun project has been deposited at DDBJ/ENA/GenBank under the accession number NBYL00000000. The version described in this paper is NBYL01000000. The BioProject accession number of the sequenced strain is PRJNA345151. The Sequence Read Archive accession number for this project is SRP102166.
